# Lipemic Serum in a Child With New-Onset Type 1 Diabetes Mellitus Presenting With Diabetic Ketoacidosis: A Case Report

**DOI:** 10.7759/cureus.45777

**Published:** 2023-09-22

**Authors:** Christina Hansa, Vimalraj Vijayakumar, Balaji Chinnasami, Subash Sundar

**Affiliations:** 1 Department of Paediatrics, SRM Medical College Hospital and Research Centre, SRM Institute of Science and Technology, Chennai, IND

**Keywords:** insulin, type 1 diabetes mellitus, children, paediatrics, hypertriglyceridemia, diabetic ketoacidosis

## Abstract

Diabetic ketoacidosis (DKA) was observed in a 12-year-old female child recently diagnosed with type 1 diabetes mellitus. A rise in triglyceride (TG) levels accompanied this. High TG levels were suspected due to the lipemic appearance of the serum. Acute pancreatitis and lipemia retinalis are potential complications for hypertriglyceridemia patients. Treating the DKA and insulin deficiency reverted the TG levels to normal without using lipid-lowering agents.

## Introduction

Diabetic ketoacidosis (DKA) is a potentially life-threatening illness that affects patients who have diabetes mellitus (DM) that is dependent on insulin, more frequently affecting those with type 1 DM. Some precipitating factors for DKA are a weak insulin regimen, insufficient monitoring, and a delay in showing up for evaluation and getting a diagnosis [1].

The diagnosis of DKA was made using the diagnostic criteria of hyperglycemia (blood sugars > 200 mg/dL), blood pH 7.3, serum bicarbonate level of < 18 mEq/L, and ketonemia (blood beta-hydroxybutyrate >/= 3 mmol/L) or ketonuria [1].

Well-known complications of DKA are cerebral edema, acute kidney injury (AKI), hypokalemia, hypoglycemia, hyperchloremic acidosis, hypochloremic alkalosis, cerebral venous sinus thrombosis, deep venous thrombosis, acute pancreatitis, and pulmonary edema [1,2]. Though any of the above can cause morbidity and mortality, the leading cause is cerebral injury [1].

A rare complication of DKA is hypertriglyceridemia (HTG), which has insulin insufficiency as its cause [3]. Triglyceride (TG) levels above 1000 mg/dL are linked to a higher risk of severe pancreatitis and cerebral edema. Acute pancreatitis and lipemia retinalis are potential complications for patients with severe HTG [3].

The relationship of HTG and DKA has been rarely described in children of South Indian heritage with newly diagnosed type 1 DM.

We describe how we successfully managed DKA and high TG levels in a 12-year-old female child using ISPAD guidelines. Informed consent was obtained from the parents.

## Case presentation

A girl who was 12 years old came to the emergency room complaining that she had been having trouble with rapid breathing for the last 24 hours. Parents gave a one-week history of visible weight loss, vomiting, increased thirst, increased urine output, burning urination, cramping muscle pains, lethargy, easy fatiguability, and inadequate oral intake. The child was not on any medications prior to the illness. According to the elicited family history, her paternal grandmother was the only other family member with (type 2) diabetes, which was well-managed with the help of oral hypoglycemic medications (OHA). There was no reported family history of dyslipidemia.

At admission, the child was alert, aware, and oriented. She weighed 25.1 kg (below the third centile for age), had a height of 133 cm (below the third percentile for age), and had a BMI of 14.19 kg/m^2^ (fifth to tenth centile). She had tachycardia, pallor, mild dehydration, acidotic breathing, mild abdominal distension, a palpable liver extending 3cm below the right coastline margin, with no indications of tenderness, and a palpable spleen extending for 2cm below the left coastal margin.

**Figure 1:**
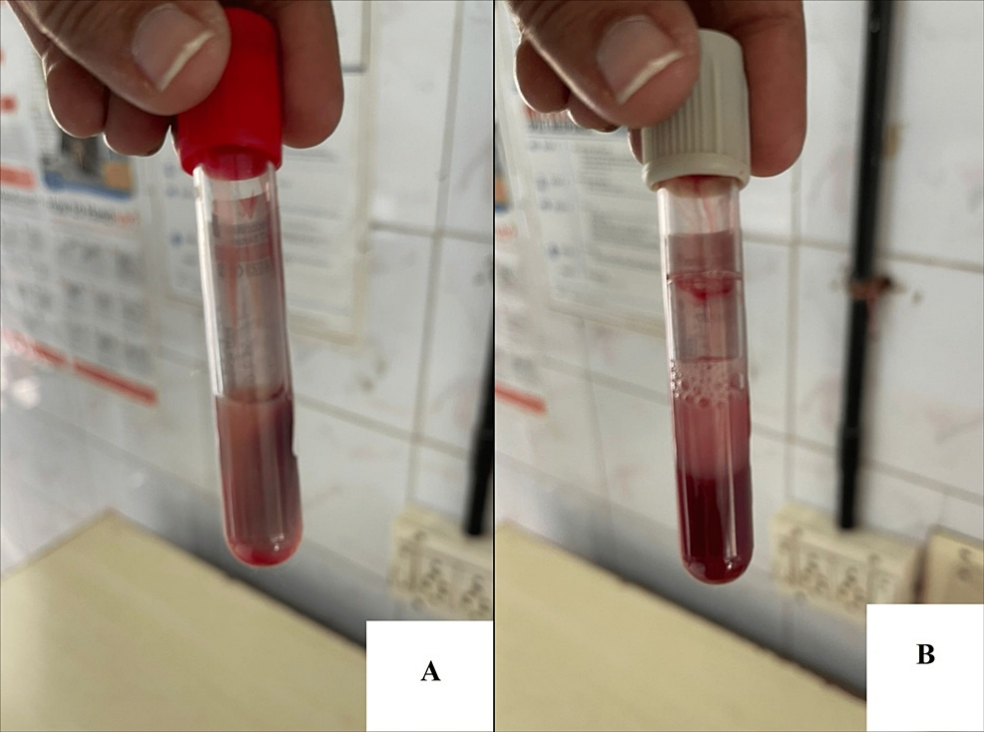
Lipemic appearance of the blood samples.

She exhibited unrecordably "high" capillary blood sugar. On taking blood samples for investigations, the blood appeared milky (lipemic) (Figures 1A, 1B). A lipid profile was sent due to the suspicion of HTG based on the lipemic appearance of the blood sample. Serum amylase and lipase results were within normal limits (serum amylase: 117 U/L and serum lipase: 54 U/L) as they were sent to screen for evidence of pancreatitis due to a known association between HTG and pancreatitis [4].

An arterial blood gas (ABG) analysis showed a blood pH of 7.27 and 17.5 mg/dL of HCO3, and a urine dipstick showed ketone ++ (ketonuria). This correlated with the clinical presentation and acute mild DKA [1].

Other significant laboratory results were blood sugar was 773 mg/dL, HbA1c was 16.7%, and TGs were 783 mg/dL. The serum sodium level of 125 mEq/L, serum potassium level of 4.4 mEq/L, serum chloride level of 90 mEq/L, and serum bicarbonate level of 18 mEq/L. The anion gap was estimated to be 17, and serum osmolality was 297 mmol/kg. The urine routine showed sugar ++ and ketone ++. Serum Amylase: 117 U/L and Serum Lipase: 54 U/L were within the normal range. Complete blood count, urea, creatinine, and liver function test were within the normal range.

Management was based on ISPAD (International Society of Pediatrics and Adolescent Diabetes) recommendations [1]. The child was initially treated with intravenous (IV) fluid bolus rehydration. Hyperglycemia control was achieved by intravenous insulin infusion at 0.05 U/kg/hr and IV fluid infusion. The insulin infusion rate was decreased to 0.025 units/kg/hr after capillary blood glucose had reduced in a desired manner for seven hours, accompanied by improvements in serum electrolytes and arterial blood pH. She was switched to subcutaneous insulin 48 hours after her DKA was controlled.

We looked for associated, although uncommon, sequelae like acute pancreatitis, peripheral venous thrombosis, pulmonary edema, and rhabdomyolysis [2]. Cerebral edema, one of the dreaded DKA side effects that raise morbidity and death, was kept an eye out for.

Genetic tests were not carried out as the child was born to healthy non-consanguineous parents. Parents were screened for the lipid profile and found to be normal.

After more tests in the lab, it was also found that she had subclinical hypothyroidism and insufficient vitamin D levels, so she was given supplements of oral thyroxine and Vitamin D. In light of the high TG levels, we obtained an ophthalmologist's opinion, and their opinion was that the fundoscopic examination did not show any signs of diabetic retinopathy or lipemia retinalis changes.

Subcutaneous insulin was adjusted during the next few days to match her daily food intake pattern to maintain a stable glycemic index throughout the day. Parents were counseled about the illness and follow-up care.

Serum TG levels were checked every week till serum TG levels normalized after which serum TG levels were checked one month later and were found to be within normal limits. Without the need for lipid-lowering medications, the child was observed on an outpatient basis until her TG levels returned to normal, which took four weeks.

## Discussion

Normal TG levels in this age group are 37-140 mg/dL [5] (Table 1). In roughly 30-50% of instances, DKA in children is accompanied by a rise in TG levels [4].

**Table 1:** Classification of hypertriglyceridemia (mg/dL) in children and adolescents Shah AS, Wilson DP: Genetic disorders causing hypertriglyceridemia in children and adolescents. Endotext [Internet] [6]

Age	Normal	Borderline	High	Very high	Severe	Very Severe
0-9 yrs	<75	≥75-99	≥100-499	≥500-999	≥1000-1999	≥2000
10-19 yrs	<90	≥90-129	≥130-499	≥500-999	≥1000-1999	≥2000

HTG, a TG level of more than 2000 mg/dL, is associated with an increased likelihood of developing acute pancreatitis [4]. Severe HTG is frequently seen during DKA and is linked to newly discovered type 1 diabetes or blamed on ineffective diabetes management with severe insulin shortage [4,7]. In our case, her HbA1c was 16.7% (normal levels: 4.5% - 5.6% [5]), which indicates poor glycemic control, and hence, intensive care was instituted.

**Figure 2:**
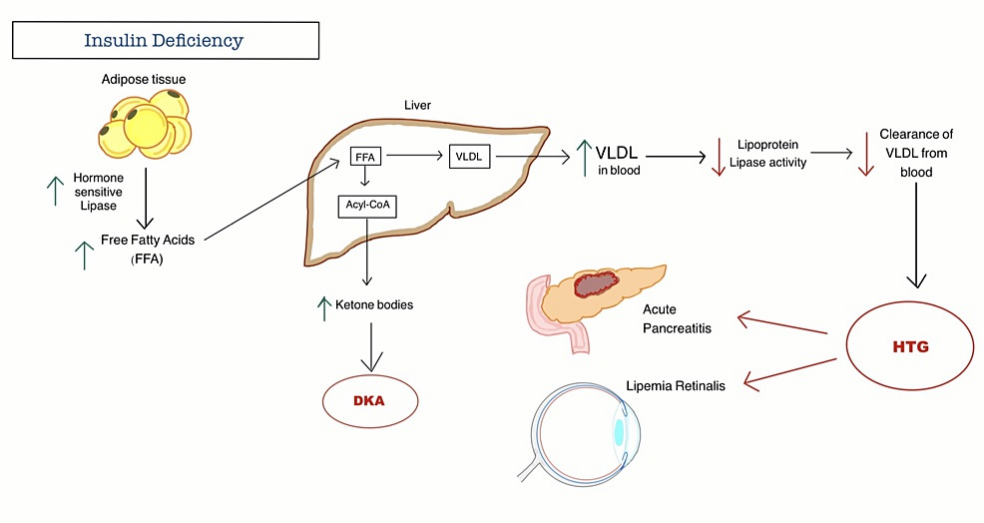
Pathophysiology of hypertriglyceridemia seen in diabetic ketoacidosis. FFA: Free fatty acid, VLDL: Very-low density lipoproteins, DKA: Diabetic ketoacidosis and HTG: Hypertriglyceridemia

The mechanism for HTG in DKA centers around the absence of insulin activity. Insulin deficiency starts the lipolysis process in adipose tissue, which causes free fatty acids (FFA) to be released and the liver to make more very low-density lipoprotein (VLDL). Insulin insufficiency also inhibits the activity of lipoprotein lipase (LPL), found in peripheral tissues, and is the enzyme responsible for the metabolism of TGs. Clearance of VLDL from the plasma is reduced due to the reduced activity of LPL, which ultimately results in the production of HTG [2,4] (Figure 2).

The disorder known as lipemia retinalis can affect as many as 23% of people who have severely elevated HTG levels (more than 2,000 mg/dL) [3]. These patients' retinal blood vessels can be shown to have changed on fundoscopy. Treatment for lipemia retinalis involves lowering TG levels to less than 500 mg/dL [3]. Therefore, a fundoscopic examination is necessary for all patients with high TG. In our case, it showed no changes consistent with lipemia retinalis.

A high percentage (14%) of HTG patients develop pancreatitis [3]. Pancreatitis is caused when the pancreatic lipase enzyme converts triglycerides into free fatty acid (FFA), which are then absorbed by the pancreas and cause inflammation [3,8]. Diabetic ketoacidosis raises the risk of pancreatitis due to HTG [3]. Increased FFA levels brought on by triglyceride hydrolysis by pancreatic lipase and insulin deprivation cause HTG-induced pancreatitis to manifest. Acute pancreatitis is brought on by high FFA levels, which destroy pancreatic acinar cells and capillary endothelium [1,9]. In our case, she was examined, evaluated, and treated for DKA, which prevented the TG levels from reaching pancreatitis thresholds.

Being aware that DKA and hypertriglyceridemia, brought on by severe insulin insufficiency, has the potential for acute cerebral oedema is essential. The proposed mechanism is cerebral blood flow is likely to be diminished by the significant dehydration of DKA and the hyper-viscosity brought on by high TG levels [2,10].

Given that insulin deficiency was the primary cause of DKA, leading to high TG levels in these cases, the standard treatment for DKA and insulin replacement reverts the high TG levels to normal levels. After four weeks of continuous monitoring, the child's TG levels returned to normal (136 mg/dl) without using TG or lipid-lowering agents.

TG and lipid-lowering agents are anti-metabolites whose safety has not been established in children [11]. Our case study provides evidence that they are not essential for treating elevated TG levels.

In view of type 1 DM being a life-long condition for the child, we worked out a nutrition plan in consultation with our pediatric nutritionist. A regular monitoring and follow up plan were made in consultation with the endocrinologist. The same was explained in detail to the parents to ensure compliance.

## Conclusions

The association of HTG and DKA in adults as a disease entity is relatively well-known and treated with established protocols. Rare reports of hypertriglyceridemia in type I insulin-dependent diabetic children with DKA have been made.

Our case emphasizes the need for a lipid profile as part of the initial diagnostic panel for all children presenting with DKA due to the known association with pancreatitis and lipemia retinalis. This presentation is of a case with very high TG levels, identified by lipemic serum, in a child with DKA treated successfully with accepted standards, and observing TG levels reverting to normal without lipid-lowering drugs.
